# Glutamate Receptor Agonists as Triggers of Neurotoxicity: Decoding Pathways of Five Neurotoxins and Potential Therapeutic Targets

**DOI:** 10.1021/acsomega.5c05841

**Published:** 2025-12-31

**Authors:** Gabriel André Turcatel, Sidnei Moura

**Affiliations:** LBIOP—Laboratory of Biotechnology of Natural and Synthetics Products—Postgraduate Program in Biotechnology, University of Caxias do Sul, Caxias do Sul 95070-560, Brazil

## Abstract

l-Glutamate (l-Glu) is one of the primary excitatory neurotransmitters in the nervous system, functioning through both ionotropic and metabotropic receptors. The release of l-Glu into the synaptic cleft, its interaction with receptors, and its reuptake are meticulously regulated by excitatory amino acid transporters. The structural similarity of various compounds to l-glutamate is crucial to their ability to interact with NMDA, AMPA, and kainate receptors. These interactions can significantly influence neural communication and function. Overstimulation of these receptors, which operate as ion channels, results in an increased level of calcium ion influx, a phenomenon known as excitotoxicity, which is often linked to neurodegeneration. Many neurodegenerative conditions are linked to both acute and chronic exposures to neurotoxins, whether they originate within the body (endogenous) or from external sources (exogenous). These neurotoxins often function as l-glutamate receptor agonists, potentially contributing to the progression of these diseases. This perspective focuses on key neurotoxins, including β-*N*-methylamino-l-alanine (l-BMAA), quinolinic acid (QUIN), domoic acid, β-*N*-oxalyl-l-α,β-diaminopropionic acid (β-ODAP), homocysteine (Hcy), and l-homocysteate, all of which exhibit complementary mechanisms of action. We will explore their structural characteristics and mechanisms through which they induce neurotoxicity. Understanding the neurotoxic mechanisms of these compounds is essential for elucidating the pathology of neurodegenerative diseases, such as amyotrophic lateral sclerosis, neurolathyrism, and amnesic shellfish poisoning. This review summarizes the findings of 64 studies to clarify these relationships involving classic events associated with neurodegeneration such as mitochondrial damage, oxidative stress, and activation of proapoptotic pathways. In summary, the distinctive properties of these neurotoxins provide valuable insights that could help in the development of future therapeutic drugs aimed at treating and alleviating the effects of neurodegenerative diseases. Understanding how these neurotoxins interact with neuronal pathways can guide researchers in designing more effective interventions.

## Graphical abstract

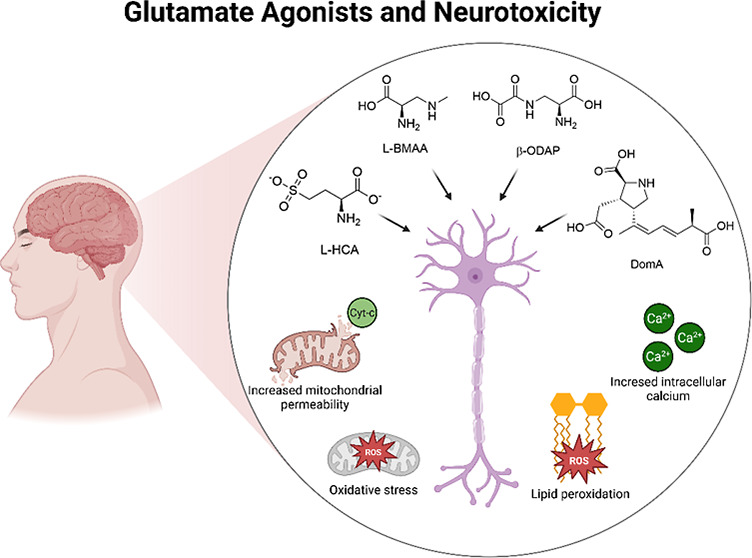

## Introduction

The excitatory role of amino acids, particularly l-glutamate (l-Glu), in the nerve cell function was first demonstrated by Curtis et al.^1^ l-Glu is one of the principal excitatory neurotransmitters in the central nervous system (CNS) and operates through two major classes of receptors: ionotropic receptors (iGluRs) and metabotropic receptors (mGluRs). The iGluRs are essential for synaptic plasticity and learning, functioning as ion channels that include *N*-methyl-d-aspartate (NMDA), α-amino-3-hydroxy-5-methyl-4-isoxazolepropionic acid (AMPA), and kainate (KA) receptors. NMDA receptors, which have a higher affinity for l-Glu, are particularly significant for calcium ion (Ca^2+^) conductivity. In contrast, AMPA and KA receptors facilitate the influx of Ca^2+^, sodium (Na^+^), and potassium (K^+^). A distinctive feature of NMDA receptors is their requirement for a coagonist, such as l-glycine or d-serine, for activation.^2,3^

In neurons, l-Glu is stored in synaptic vesicles located at the presynaptic terminal. Upon the arrival of an action potential, it is released into the synaptic cleft, where it interacts with receptors on postsynaptic neurons, leading to an influx of ions^4,5^ (Figure 1). Under normal physiological conditions, l-Glu is cleared from the synaptic cleft by excitatory amino acid transporters (EAATs) found in the membranes of astrocytes and neurons. Within astrocytes, l-Glu is converted to glutamine by the enzyme glutamine synthetase. This glutamine is then transported from astrocytes to neurons via a sodium-coupled neutral amino acid transporter (SNAT), where it is reconverted to l-Glu by glutaminases for reuse as an excitatory neurotransmitter.^2,5,6^ There are five EAATs, with EAAT1 and EAAT2 predominantly found in astrocytes, macrophages, and oligodendrocytes. EAAT3, primarily expressed in neurons, facilitates the transport of both l-Glu and cysteine, serving as a protective mechanism against oxidative stress in the CNS.^2,7^ EAAT4 is located in postsynaptic structures, while EAAT5 is restricted to rod photoreceptors in the retina.^6^

**Figure 1:**
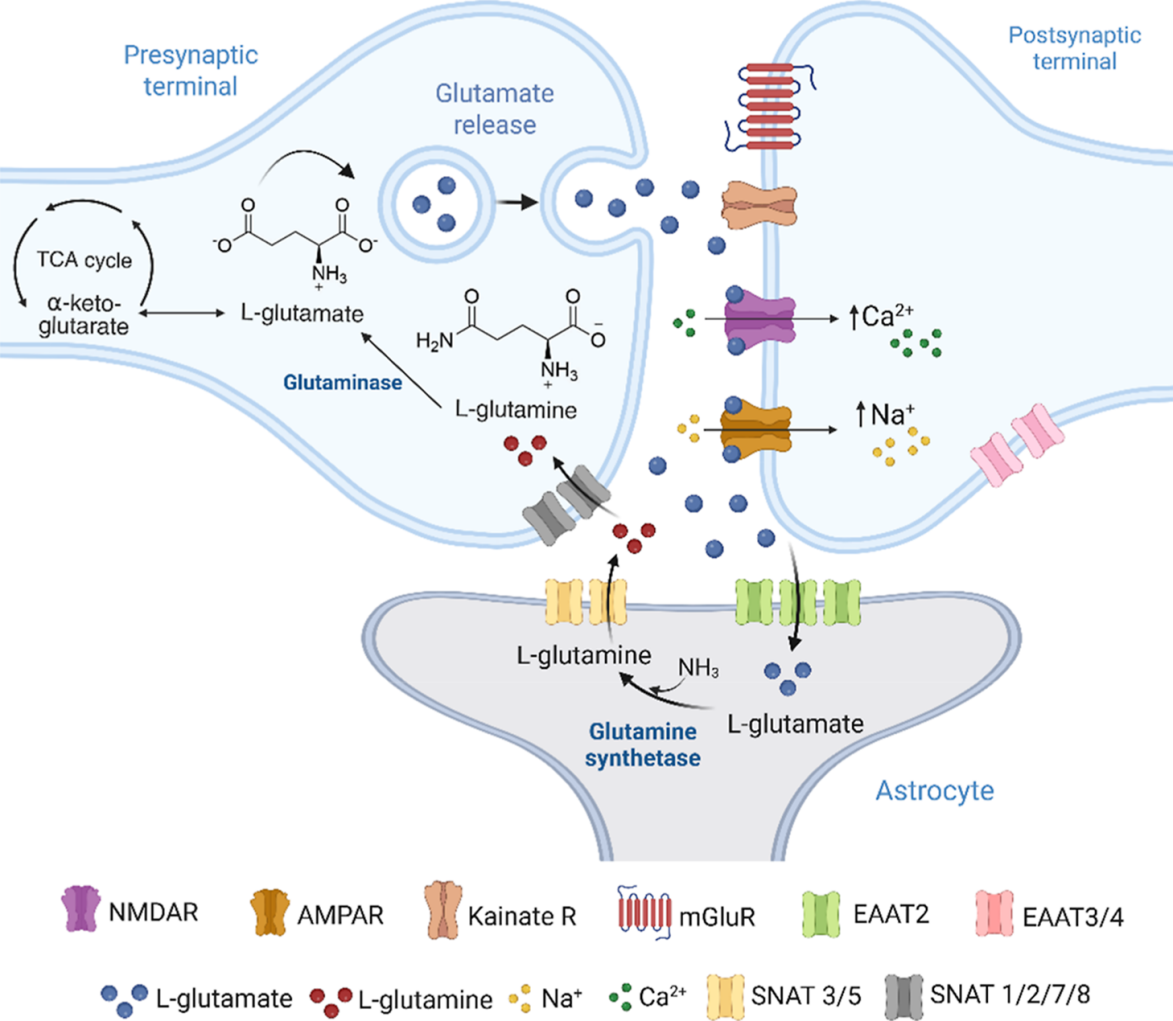
l-Glu cycle. l-Glu is synthesized in the presynaptic neuron from α-ketoglutarate. Upon release into the synaptic cleft, l-Glu interacts with various postsynaptic receptors (NMDAR, AMPAR, KA, and mGluR), modulating neuronal excitability through the influx of Na^+^ and Ca^2+^ ions. The removal of extracellular glutamate is mediated by EAATs (EAAT2 in astrocytes and EAAT3/4 in neurons). In astrocytes, glutamate is converted to l-Glu by glutamine synthetase, which is then transported back to neurons SNAT, completing the cycle. This mechanism is essential for maintaining excitatory neurotransmission and synaptic homeostasis in the CNS.

Excitotoxicity occurs when there is an excessive accumulation of excitatory amino acids in the extracellular space, leading to hyperstimulation of ionotropic receptors in neural cells. This hyperstimulation results in increased intracellular calcium concentrations, elevated levels of reactive oxygen species (ROS), and activation of metabolic pathways and catalytic enzymes that can induce apoptosis.^8^ This action is closely associated with various neurodegenerative diseases and can be triggered by factors such as increased synthesis of l-Glu, decreased expression of EAATs, structural changes that impair l-Glu reuptake, and the presence of neurotoxic compounds that interact with iGluRs, including other excitatory amino acids.^6^

The structural similarity between potentially excitotoxic compounds and l-Glu is critical for facilitating the stimulation of iGluRs. The three-point receptor system theory, proposed by Curtis et al.,^1^ posits that receptors possess two to three charged sites that interact with the ionized groups of amino acids. Endogenous molecules like l-homocysteate (l-HCA) and tryptophan metabolites, along with exogenous compounds such as domoic acid (DomA),^9^ β-*N*-methylamino-l-alanine (l-BMAA),^10,11^ and β-*N*-oxalyl-l-α,β-diaminopropionic acid (β-ODAP),^12^ have been identified as l-glutamate receptor agonists. These substances can induce excitotoxicity, which can be harmful to nerve cells.^2,13^

Numerous neurodegenerative disorders in humans have been linked to both acute and chronic exposures to neurotoxins, particularly excitatory amino acids, which can interact with l-Glu receptors.^6^ In this line, this review aims to describe key compounds known to act as l-Glu receptor agonists at ionotropic receptors, including l-BMAA, quinolinic acid (QUIN), DomA, β-ODAP, and l-HCA. We will explore the neurotoxic mechanisms triggered by each of these compounds and discuss their significance within the context of neurodegenerative processes. Ultimately, this paper serves as a guide to understanding the role of exogenous environmental contaminants in neurodegenerative diseases, potentially facilitating the development of new therapeutic compounds.

### β-*N*-methylamino-l-alanine

l-BMAA is a nonproteinogenic amino acid first identified by Vega and Bell in 1967 ^14^ from the seeds of *Cycas circinalis*. It is also recognized as a secondary metabolite produced by certain cyanobacteria, particularly from the genera *Nostoc*, *Nodularia*, *Calothrix*, and *Anabaena.* ^15,16^ Additionally, l-BMAA is synthesized by eukaryotic microalgae and widely distributed bacteria, such as *Paenibacillus* spp.^17^

The neurotoxic potential of l-BMAA was later emphasized in studies by Spencer et al.^18^ and Cox et al.^19^ who linked the compound to elevated rates of neurodegenerative diseases, including amyotrophic lateral sclerosis (ALS) and the ALS–Parkinson’s dementia complex (ALS/PDC) among the Chamorro people of Guam in the North Pacific. Research indicates that chronic exposure of the local population to this compound primarily occurred through the consumption of cycad seed flour, which contains symbiotic cyanobacteria of the genus *Nostoc*.^20^ Additionally, exposure was linked to the consumption of bats (*Pteropus mariannus mariannus*), a local delicacy that feeds on the seeds of *C. circinalis,* today called *Cycas micronesica Hill.* ^21–24^ Extensive studies on the effects of l-BMAA on the CNS have been conducted using cell cultures and in vivo models, revealing various mechanisms of neurotoxicity and the compound’s ability to induce neurodegeneration (see Table 1).

**Table 1:** Mechanisms of Action and Neurotoxic Effects of l-BMAA in Various In Vitro and In Vivo Models

specie/cell culture	concentration	duration	key effects	reference
zebrafish (Danio rerio)	5 μg mL	10 days	seizures and abnormal formation of the spinal axis	^76^
Artemia salina	300 μg mL	24 h	elevated mortality rates	^76^
Nassula sorex	0.05 μg mL	72 h	elevated mortality rates	^76^
Drosophila melanogaster	25 mM	1–5 days	alteration in the function of postsynaptic cells, resulting in a decrease in wingbeat frequency	^77^
Delphinus delphis			dystrophic neurites, neurofibrillary tangles, and the formation of β-amyloid plaques	^67^
Macaca fascicularis		30 days	conduction deficits in motor pathways, accompanied by muscle weakness, loss of muscle mass, and a stooped posture	^18^
Sprague–Dawley mice	500 ug	10 min	muscle spasms, hyperactivity, and facial tremors, accompanied by elevated intracellular Ca^2+^ levels	^78^
Sprague–Dawley mice	5 mM	50 seg	elevated intracellular Ca^2+^ levels	^11^
Wistar mice microglial cell culture	0.1–3 mM	24–48 h	enhanced release of lactate dehydrogenase, influx of Ca^2+^, and production of ROS	^79^
Swiss Webster mice mixed cortical	3 mM	1 h	inhibition of the cystine/glutamate antiporter (system Xc^–^), leading to oxidative stress, elevated extracellular glutamate levels, and excitotoxicity	^80^
primary neuronal stem cell culture	1–3 mM	24 h	production of elevated levels of ROS and oxidative stress, resulting in DNA damage	^81^
primary human astrocytes	0.38 μM and 5.45 μM	24 h	dose-dependent increases in LDH, elevated Ca^2+^ influx, oxidative stress, excitotoxicity, reduced cell proliferation, and cell death consistent with neurodegenerative processes	^82^
MRC-5, human lung fibroblast cell line	31.25 nM	16 h	incorporation of l-BMAA into protein synthesis and subsequent release of this compound following protein hydrolysis	^83^
male C57/BL mice	3.8 mg/kg body	8 h	accumulation of l-BMAA in gray matter, with lower levels observed in white matter tracts	^29^
mice, dissociated spinal cord culture	30, 100, 300, and 1000 μM	30 min –24 h	dose-dependent activity of lBMAA leads to fragmentation of cell bodies, stimulation of AMPA and KA receptors, increased intracellular Ca^2+^ levels, and the formation of ROS	^84^
mice, Sprague–Dawley	10 mM	24 h	reduced levels of taurine, serine, and glycine in brain tissue	^85^
mice, mixed cortical cell cultures containing neurons and astrocytes	0.1–10 mM	24 h	concentrations greater than 1 mM induced neuronal death. In treatments below 1 mM, an increase in Ca^2+^ influx, oxidative stress, and hyperstimulation of NMDA and mGluR5 receptors were observed	^86^

The neurotoxicity of l-BMAA is linked to its chemical structure, which allows for a competitive interaction with neuronal NMDA receptors and mGluRs. The primary mechanism underlying l-BMAA’s neurotoxic effects is the hyperstimulation of NMDA receptors, which activates a signaling cascade that opens ion channels and increases Ca^2+^ influx. This excessive calcium influx disrupts cellular homeostasis, ultimately leading to neuronal damage and degeneration.^10,25–27^ The intracellular elevation of Ca^2+^ negatively impacts mitochondrial metabolism by increasing mitochondrial membrane permeability and promoting the formation of pores that allow the release of proapoptotic proteins. This compromise of the respiratory chain reduces ATP synthesis and enhances the production of free radicals and ROS. Collectively, these factors contribute to oxidative stress, which is a critical element associated with neurodegenerative conditions. This oxidative stress disrupts cellular metabolism and organelle function, as illustrated in Figure 2.^10,14,28^

**Figure 2:**
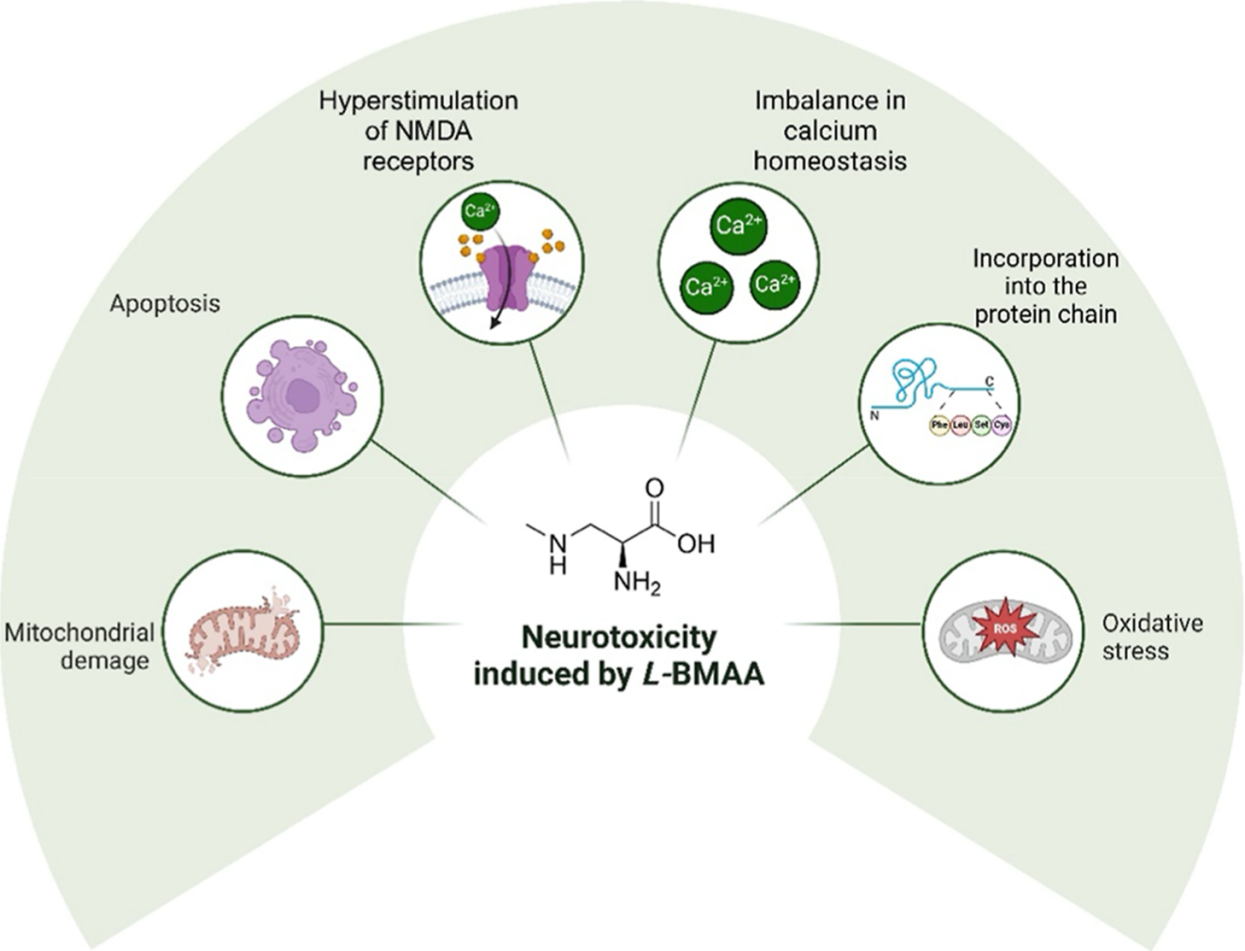
l-BMAA toxicity. Key mechanisms of l-BMAA-induced neurotoxicity implicated in the pathophysiology of neurodegenerative diseases.

l-BMAA’s ability to disrupt protein synthesis in eukaryotic cells represents another critical mechanism of its toxicity. The arrangement of the amino and carboxyl groups in the structure of l-BMAA facilitates the formation of peptide bonds, allowing it to be incorporated into amino acid chains during protein synthesis. This incorporation can lead to improper folding of tertiary structures and the accumulation of toxic oligomeric clusters that resist proteolytic degradation. Consequently, these misfolded proteins can disrupt cellular function and contribute to neurodegenerative processes.^15,29,30^ The association of this compound with proteins was demonstrated by Murch et al.,^16^ who evaluated the concentrations of free and protein-bound l-BMAA in brain tissue. Their study underscored the importance of this compound’s interaction with proteins in understanding its neurotoxic effects. Specifically, Cox et al.^19^ and Murch et al.^16^ reported approximately 6 μg/g of free amino acid in the frontal cortex tissue of Chamorro patients affected by the ALS/PDC complex. In contrast, they found an average concentration of 627 μg/g in the protein-associated fraction, indicating the significant biomagnification of this compound. These findings reinforce the link between chronic exposure to this toxin and the development of neurodegenerative diseases. The protein-bound fraction acts as an endogenous reservoir, gradually releasing l-BMAA over time and contributing to sustained neurotoxic effects.

### Quinolinic Acid

Pyridine-2,3-dicarboxylic acid, commonly known as QUIN, is an endogenous agonist of NMDA receptors. It is produced through the enzymatic oxidation of l-tryptophan (l-Trp) via the kynurenine pathway (KP) in microglial cells and macrophages (see Figure 3). The KP plays a crucial role in regulating sleep, behavior, and cognition while generating intermediates that possess both neurotoxic and neuroprotective properties. Among these metabolites, QUIN is particularly significant in terms of bioactivity within the CNS, especially affecting pyramidal neurons in the hippocampus.^31^

**Figure 3:**
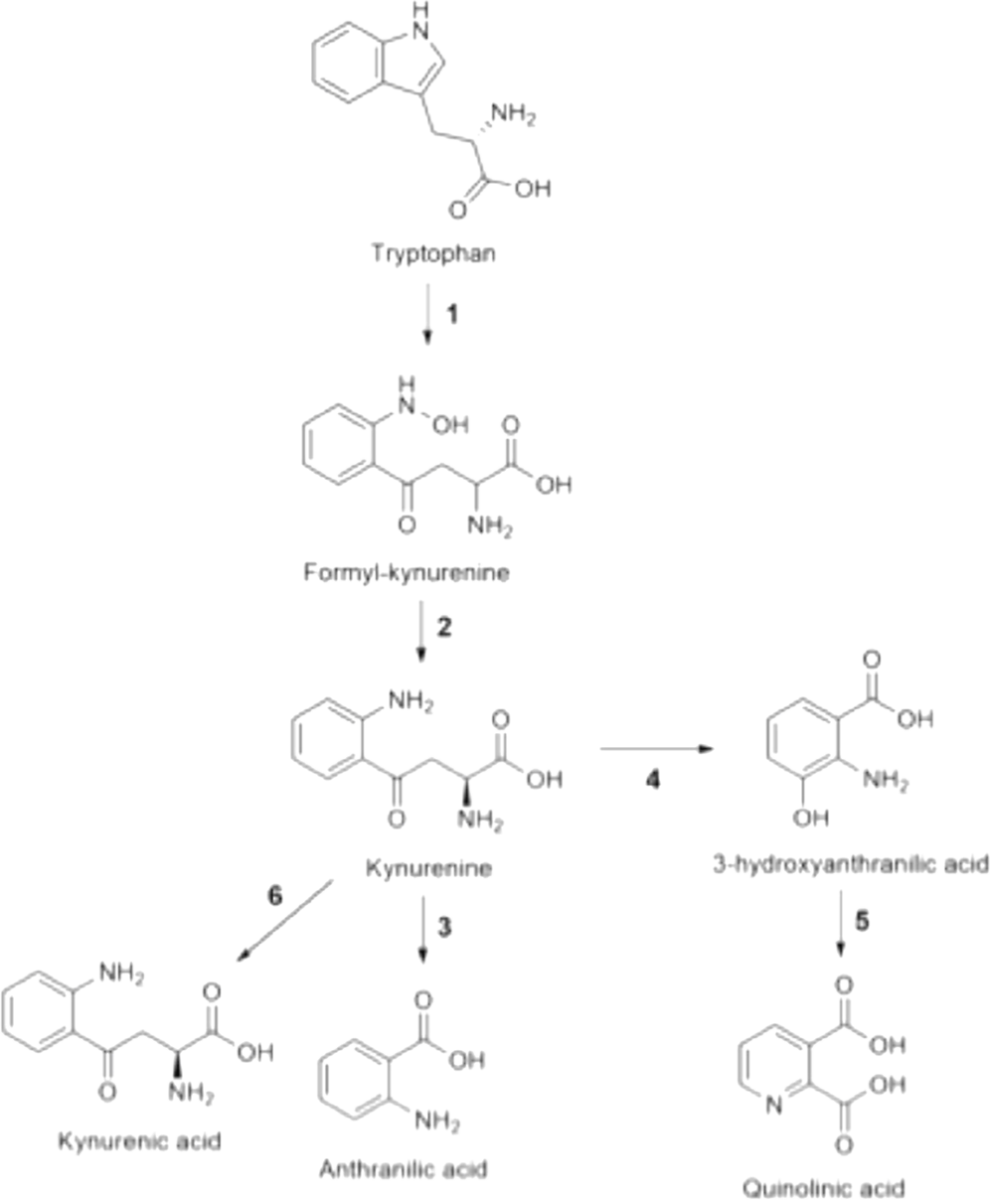
KP. l-trp degradation through the KP and the formation of neuroactive compounds in mammalian cells. 1: TDO or IDO, 2: kynurenine formamidase, 3: kynureninase, 4: kynurenine 3-monooxygenase, 5:3-hydroxyanthranilic acid 3,4-dioxygenase, 6: kynurenine aminotransferase I, II or III.

The pathway begins with the conversion of l-Trp to kynurenine, facilitated by the enzyme tryptophan-2,3-dioxygenase (TDO), which is found in neurons, astrocytes, and endothelial cells, or indoleamine-2,3-dioxygenase (IDO), which is expressed in microglial cells and astrocytes. Depending on physiological conditions, kynurenine can be further converted to kynurenic acid (KYNA), a neuroprotective NMDA receptor antagonist, or 3-hydroxykynurenine (3-HK). The latter serves as a precursor for the synthesis of QUIN and picolinic acid (PIC), Figure 3.^31–33^ The KP is compartmentalized based on cellular physiology: 3-HK and its derivatives are primarily synthesized in microglial cells, while KYNA is produced in astrocytes.^32,34^

The initial evidence of kynurenine’s neurotoxicity was documented in 1981 by Stone and Perkins, who observed convulsions in mice following the administration of QUIN into the cerebral ventricles. This research established this compound as an excitatory endogenous molecule that acts on NMDA receptors in CNS neurons.^35^

Under physiological conditions, the concentration of kynurenine in the brain and cerebrospinal fluid typically ranges from 50 to 100 nM, where it acts as a substrate for the synthesis of nicotinamide adenine dinucleotide (NAD^+^). However, concentrations exceeding 150 nM are associated with pathological and inflammatory processes, often linked to an increased expression of IDO and neurotoxicity. This neurotoxicity can arise through multiple mechanisms, as illustrated in Figure 4.^8^ During neuroinflammatory processes, approximately 95% of brain l-Trp is catabolized via the KP in glial cells, resulting in the formation of neuroactive metabolites. This catabolism is driven by the upregulation and increased activity of IDO, which is stimulated by proinflammatory cytokines such as interferon-gamma and tumor necrosis factor-alpha, as well as by bacterial lipopolysaccharides.^35^

**Figure 4:**
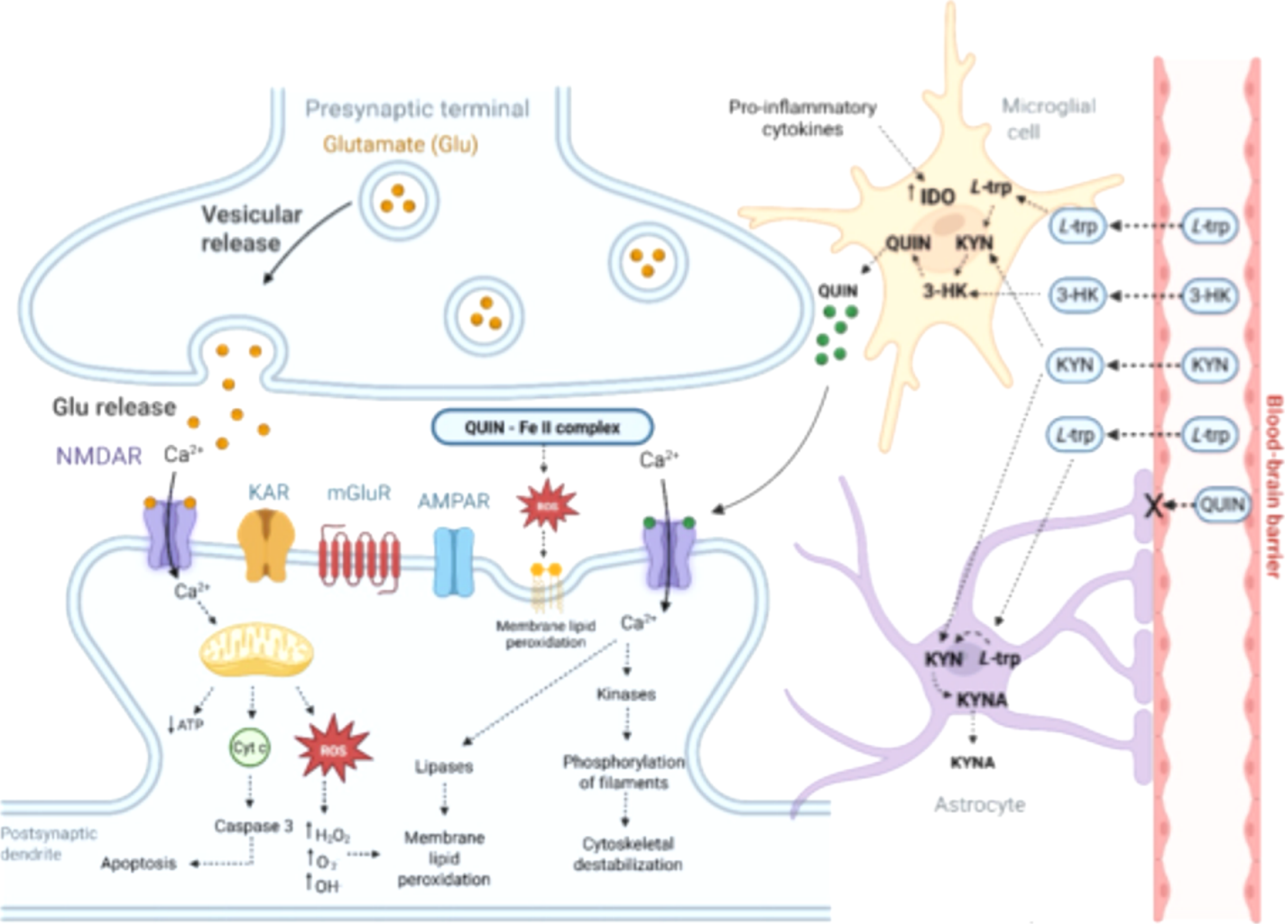
QUIN neurotoxicity. Overview of the multiple mechanisms of neurotoxicity associated with quinolinic acid.

While the blood–brain barrier (BBB) is impermeable to QUIN, it permits the passage of peripheral l-Trp and metabolites such as KYN and 3-HK. These compounds can be absorbed and metabolized by glial cells, leading to the accumulation of toxic metabolites in the CNS.^8,33^ Consequently, inflammatory reactions significantly influence the dynamics of the KP both peripherally and centrally, potentially causing an imbalance in the production and levels of neuroactive KP metabolites in the brain.^31,36,37^ The accumulation of QUIN can occur both intracellularly and extracellularly. Once synthesized, this molecule can be readily secreted from cells without a reuptake mechanism, allowing it to accumulate in the surrounding environment.^35^

One of the mechanisms of neurotoxicity associated with chronic exposure to this molecule under neuroinflammatory conditions is the elevation of intracellular Ca^2+^ concentration. Similar to l-BMAA, the hyperstimulation of NMDA receptors by QUIN results in increased influx of this cation, which subsequently induces mitochondrial dysfunction and destabilizes the cytoskeleton. The excess calcium in the mitochondrial membrane triggers the formation of pores, compromising the respiratory chain and increasing membrane permeability. This disruption leads to the production of free radicals, such as hydroxyl radicals (^•^OH) and superoxide anions (O_2_^•–^), as well as ROS like hydrogen peroxide (H_2_O_2_). Moreover, elevated calcium levels (Ca^2+^) play a crucial role in releasing mitochondrial contents, like cytochrome C. This release can activate caspases, leading to cell death through apoptosis.^8,38^ Cytoskeletal destabilization occurs through the activation of calcium-dependent protein kinases (PKCα/βII), which enhance the phosphorylation of serine residues in intermediate filament proteins of the cytoskeleton in both neurons and astrocytes. Neurofilaments are synthesized in the cell body of neurons and transported along the axon via axonal transport, where they integrate into the cytoskeleton to maintain cellular rigidity and stability. An imbalance in phosphorylation levels can slow axonal transport, leading to aggregation of neurofilaments. This aggregation disrupts the organizational structure of the cytoskeleton, impairs cellular function, and inhibits the reuptake of extracellular glutamate.^39^

Meanwhile, a second mechanism contributing to the toxicity of this compound is initiated by the Fenton reaction, which occurs when H_2_O_2_, resulting from mitochondrial damage, interacts with intracellular Fe^2+^. This reaction generates ^•^OH, which can cause significant damage to cellular DNA and lead to peroxidation of membrane lipids, ultimately increasing cellular permeability. Additionally, these radicals can form through coordination complexes between QUIN and Fe^2+^, further exacerbating lipid peroxidation and oxidative stress. Elevated levels of intracellular ROS can enhance the release of glutamate at nerve terminals, thereby amplifying its primary mechanism of excitotoxicity.^31,34^ Furthermore, increased concentrations of quinolinic acid and glutamate can inhibit the synthesis of the neuroprotective compound KYNA, representing an additional mechanism of toxicity.^36^ Elevated levels of this compound have been observed in patients with neurodegenerative diseases such as Alzheimer’s disease, ALS, Parkinson’s disease, and Huntington’s disease. Comparative studies of postmortem brain tissue have revealed significantly higher neuronal levels of quinolinic acid in patients with Alzheimer’s and Huntington’s diseases compared to those in control groups. Additionally, these studies have shown increased immunoreactivity for QUIN and IDO in glial cells located near amyloid plaques, further highlighting the potential role of quinolinic acid in neurodegenerative processes.^31,32,35^ Moreover, elevated levels of this metabolite have also been detected in the cerebrospinal fluid of patients with ALS compared to that in healthy individuals.^46^ In addition to immunological stimuli in the CNS, viral infections such as HIV-1 can also activate the KP by increasing levels of 3-HK and quinolinic acid, which may surpass the concentrations of the neuroprotective compound KYNA.^31,35^

### Domoic Acid

This natural compound, produced as a secondary metabolite by certain species of microalgae, is responsible for synthesizing toxic metabolites, whose functions remain largely unknown. DomA is a cyclic amino acid generated by diatoms of the genus *Pseudonitzschia* and is considered one of the most significant marine toxins concerning public health.^26,40,41^ It was first described by Takemoto and Daigo in 1958 after being isolated from the red alga *Chondria armata* along the southern coast of Japan, where extracts of this alga were traditionally used to treat intestinal parasites at doses of approximately 20 mg.^40,42^ The synthesis of this toxin varies with environmental conditions; however, it is known to bioaccumulate in fish, such as anchovies, and filter-feeding mollusks, such as mussels. Humans are primarily exposed to this compound through the consumption of contaminated shellfish. Its structural similarity to KA and l-Glu arises from the presence of three carboxylic groups, enabling it to interact with AMPA, KA, and NMDA receptors.^40,42,43^

In 1987, Canada experienced the largest documented outbreak of this marine toxin poisoning, resulting in 153 cases and four fatalities due to the consumption of contaminated blue mussels (*Mytilus edulis*). The most affected individuals were elderly patients or those with pre-existing conditions, such as diabetes, renal failure, and hypertension, who exhibited more severe clinical symptoms. Reports indicated that gastrointestinal symptoms, including vomiting and nausea, began 4–5 h after exposure and rapidly progressed to headaches, memory loss, mental confusion, and disorientation. Following this outbreak, DomA poisoning became known as amnesic shellfish poisoning (ASP).^44,45^ In the most severe cases of contamination, the estimated concentration ingested was approximately 290 mg. However, 1 week after the onset of symptoms, this compound was not detected in the blood or cerebrospinal fluid of patients, likely due to its hydrophilicity and short plasma half-life. In response to this public health concern, Canada and other countries have established a maximum allowable limit for this toxin in shellfish, which is set at 20 μg/g. However, doses that do not induce acute neurotoxicity may have long-term effects due to chronic DomA exposure.^42,46^

The mechanism of toxicity associated with acute exposure to this toxin is primarily due to its high affinity for iGluRs, particularly AMPA and KA types. When these receptors are activated in neurons and astrocytes, there is an increase in Ca^2+^ influx, leading to the generation of ROS, lipid peroxidation, and ultimately necrosis. Elevated concentrations of this compound result in the hyperstimulation of these receptors, which can induce the release of vesicular glutamate into the synaptic cleft. This excess glutamate then binds to NMDA receptors on the postsynaptic neuron, further amplifying the initial neurotoxic mechanism.^26,43^ This was investigated by Radad et al.^9^ also, who found that after 4 days of exposure to concentrations ranging from 0.1 to 100 μM, mouse dopaminergic neurons exhibited significant morphological changes, including cellular dimorphism and reduced neurite length, compared to neurons that received cotreatment with antagonists of AMPA and KA receptors.

This compound has a low absorption in the gastrointestinal tract. Due to its highly polar chemical structure, approximately 75% is eliminated via urine without requiring metabolic conversion, with a half-life of about 20 min. Three groups are particularly susceptible to its toxicity: the elderly, who have diminished antioxidant defenses at the neural level; individuals with chronic kidney disease, where reduced renal clearance prolongs exposure to the toxin; and those with conditions that compromise the integrity of the BBB.^26,40,43^ Histopathological analyses of the brains of rodents exposed to 5 mg/kg per day of this compound for 64 days revealed acute brain damage and changes characteristic of neurodegenerative processes. These changes included vacuolization of the cytoplasm in both neurons and astrocytes, as well as cellular swelling due to ion influx.^43^

Some studies have suggested that this toxin does not have teratogenic effects; however, exposure during fetal development in rodents has been associated with hippocampal lesions, seizure disorders, and persistent behavioral changes, likely linked to compromised integrity of the BBB.^40^

Giordano et al.^45^ investigated the apoptosis induced by this compound through the formation of ROS. Their study demonstrated an increase in oxidative stress markers in rodent neurons exposed to 0.1 μM of the toxin, including elevated levels of oxidized glutathione, the release of mitochondrial cyt-c, and the activation of caspase-3. Since the poisoning incident in 1987 and the subsequent regulation of DomA in food, no further cases of acute poisoning in humans have been reported. However, preclinical studies indicate that even chronic exposure to levels below the established residual limit can still cause mild damage to neural cells.^42^

X-ray crystallography has been essential in understanding the molecular mechanisms and structure–activity relationship of the interaction between iGluRs and DomA. The Protein Data Bank presents precise details on how DomA binds to different l-Glu receptor subunits, especially KA receptors.^47^ Among the most relevant structures is the complex formed between DomA and the S1S2 domain of the GluR6 subunit of the KA receptors. In addition to aiding in decoding the mechanisms of domoic acid-mediated excitotoxicity, the technique can also be applied in molecular modeling studies for the development and evaluation of iGluR antagonists.

### β-*N*-oxalyl-l-α,β-diaminopropionic Acid

The neurotoxin β-ODAP, also known as dencichine, is a nonproteinogenic excitatory amino acid found in the seeds of the legume *Lathyrus sativus*. This plant has been utilized since the Neolithic period and plays a significant role in the diets of developing countries in Asia and Africa due to its high protein content (28–49%) and its resilience in adverse conditions, such as drought, excessive rainfall, high temperatures, and poor soil fertility. In 1996, approximately 2000 members of a tribe in Ethiopia suffered from long-term excessive consumption of *L. sativus*, resulting in the development of neurolathyrism. This neurodegenerative disease is characterized by the loss of axons in the lumbar spinal cord, which can progress to impaired mobility without support and, in severe cases, lead to irreversible paralysis of the lower limbs.^12,48,49^ Initial research indicated that neurolathyrism is a consequence of the prolonged consumption of *L. sativus* seeds containing up to 1% of this neurotoxin.^56^ This compound is present throughout all parts of the plant, with the highest concentrations found in the leaves. It is believed that this compound plays a role in zinc transport and acts as a protective molecule during photosynthesis, particularly under conditions of high radiation intensity.^50^

Since the 1960s, numerous studies have investigated the role of β-ODAP in its interaction with l-Glu receptors and its effects on neuronal signaling. Its ability to bind to AMPA receptors results in an increased influx of Ca^2+^, which subsequently enhances the expression of the β-1 integrin on the cell surface. This increase in expression leads to greater phosphorylation of actin units, ultimately disrupting the structure of microfilaments and affecting cytoskeletal dynamics.^12,51^ In vivo assays conducted on spinal cord neurons of frogs demonstrated that this toxin induces neuroexcitation and inhibits the glutamate reuptake system, revealing a specific affinity of this neurotoxin for AMPA receptors.^52^

Excitotoxicity alone does not account for the neuronal damage and pathology associated with long-term exposure to this compound. In normal physiological processes, l-Glu is released into the synaptic cleft, where it binds to iGluRs. This interaction induces depolarization of the cell membrane, leading to an increased level of influx of Ca^2+^. Subsequently, l-Glu is cleared from the synaptic cleft by EAATs located on the membranes of astrocytes and neurons. In contrast, nonproteinogenic amino acids such as β-ODAP and l-BMAA do not undergo the same reuptake mechanism. As a result, they can persist in the synaptic cleft, contributing to a prolonged Ca^2+^ influx, which may exacerbate neuronal damage and pathology. This distinction highlights the unique risks associated with chronic exposure to these nonproteinogenic compounds.^49^

Oxidative stress plays a significant role in the etiology of neurolathyrism and involves several mechanisms. Notably, this stress is linked to interactions with mitochondrial complex I, which leads to the generation of ROS. Furthermore, oxidative stress inhibits the activity of crucial reducing enzymes such as catalase and glutathione peroxidase, which are essential for maintaining cellular redox balance. When cell lysis occurs, cytosolic l-Glu is released, leading to excitotoxicity in the neighboring cells. This process initiates a feedback loop known as the glutamatergic cycle, where increased glutamate levels further stimulate excitotoxic effects. This cycle significantly contributes to neuronal degeneration, underscoring the interconnectedness of oxidative stress and excitotoxicity in the progression of neurolathyrism. Understanding these mechanisms can provide insights into potential therapeutic strategies to mitigate neuronal damage associated with this condition.^48,49,53^

### Homocysteine and l-Homocysteic Acid

l-Homocysteic acid (l-HCA), an endogenous nonproteinogenic amino acid present in the CNS, is formed through the oxidation of homocysteine (Hcy). Initial studies suggest that l-HCA’s structural similarity to l-Glu may enable it to disrupt synaptic transmission and alter neurotransmitter function, particularly within the glutamatergic system. Notably, this compound demonstrates a high affinity for NMDA receptors and has the potential to induce excitotoxicity at levels comparable to those of l-Glu. This raises concerns regarding l-HCA’s role in modulating synaptic activity and its implications for neuronal health and function.^54–56^ Understanding these interactions is crucial for exploring the potential impacts of this molecule on neurophysiological processes and its contribution to neurological disorders.^57–60^

One of the earliest pieces of evidence supporting the mechanism of action of l-HCA emerged from studies demonstrating acute excitotoxicity in the retinas of chicken embryos, which mirrored the effects induced by NMDA.^59^ Additionally, research by Pullan et al.^61^ revealed that this compound has a significantly higher affinity for NMDA receptors compared to other iGluRs, with its binding affinity for NMDA receptors being approximately 10 times greater than that of AMPA and kainic acid receptors. Furthermore, other sulfur-containing amino acids, such as cyclic acid and D-HCA, exhibited partial selectivity for NMDA receptors. These findings emphasize the importance of l-HCA in modulating glutamatergic signaling and its potential implications for excitotoxicity in the nervous system.^59^

Recent studies have also investigated the potential excitotoxic effects of Hcy. According to Kruman et al.,^62^ homocysteine can interact with NMDA receptors, resulting in several detrimental cellular outcomes, including DNA damage, caspase activation, and increased levels of mitochondrial free radicals. These events culminate in nuclear disintegration and apoptosis of rodent hippocampal neurons in vitro, contributing to the pathogenesis of neurodegenerative diseases and stroke. Notably, plasma homocysteine levels tend to rise with age, with concentrations exceeding 15 μmol/L linked to cognitive decline, dementia, and Alzheimer’s disease.^63,64^

Additionally, similar to other l-Glu receptor agonists, homocysteine concentrations above 100 μM can enhance calcium influx, particularly in the presence of cofactors, such as glycine. This increase in Ca^2+^ leads to a cascade of events, including the generation of free radicals that promote lipid peroxidation in nerve and endothelial cells—a hallmark of neurodegenerative processes. These findings underscore the significance of Hcy in neurotoxicity and its potential role in the progression of neurological disorders.^62,65,66^

All compounds described exhibit structural features analogous to those of l-Glu and can interact with its receptors through ionic interactions between charged groups. The dashed lines and charge symbols (±) in Figure 5 illustrate the electrostatic mimicry between the compounds and the receptor binding site. These compounds possess the ability to aberrantly activate l-Glu receptors, resulting in excessive Ca^2+^ ion influx into neuronal cells, oxidative stress, and ultimately neurodegeneration.^1,2^

**Figure 5:**
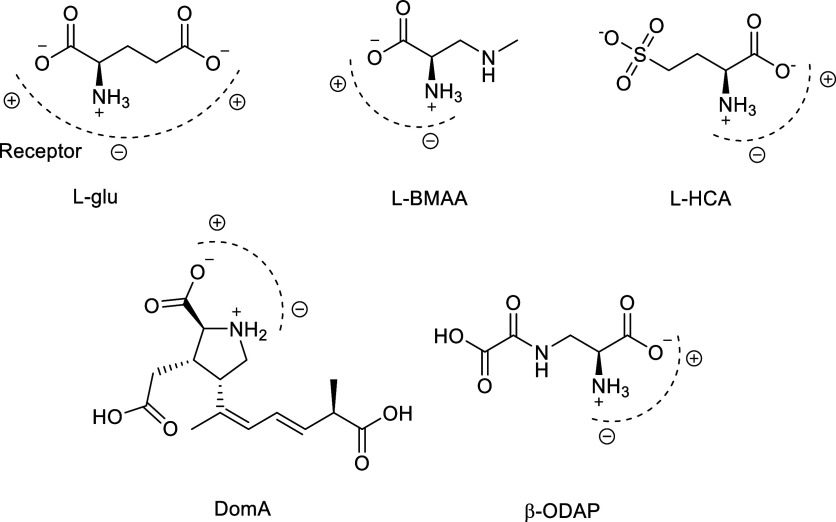
Electrostatic mimicry of neurotoxins. “Three-pronged” dipolar binding system for glutamate and other excitatory compounds at their respective receptor.

The structures of these compounds contain essential functional groups for receptor recognition, such as carboxylate and protonated amino groups, enabling them to bind to the same sites physiologically occupied by l-Glu.^1^ Understanding the structure–activity relationship of these analogues is crucial not only for elucidating the mechanisms of neurotoxicity but also for guiding the development of therapeutic antagonists or strategies to mitigate environmental exposure. Table 2 summarizes the main experimental findings related to the neurotoxic effects of the l-Glu receptor agonists included in this review. It presents the experimental models used, the concentrations administered, and the biochemical and cellular effects observed, with a focus on processes, such as oxidative stress, Ca^2+^ influx, and mitochondrial dysfunction. These effects lead to reduced cell viability and the induction of cell death processes, including necrosis and apoptosis, consistent with the pathological mechanisms seen in neurodegenerative diseases.

**Table 2:** Summary of the Primary Mechanisms of Neurotoxicity Associated with l-Glu Receptor Agonists, Including the Cellular or Animal Models Used, Concentrations Tested, and Main Effects Observed

compound	model/cell culture	concentration	key findings	reference
l-BMAA	primary human astrocytes	0.38 μM and 5.45 μM	dose-dependent increases in LDH, elevated Ca^2+^ influx, oxidative stress, excitotoxicity, reduced cell proliferation, and cell death consistent with neurodegenerative processes	^82^
	MRC-5, human lung fibroblast cell line	31.25 nM	incorporation of l-BMAA into protein synthesis, followed by its release upon protein hydrolysis	^83^
QUIN	male Wistar rats	120 nmols	dose-dependent damage to hippocampal cells induced by lipid peroxidation	^87^
	adult male Wistar rats	60–480 nmol/μL	oxidative stress disrupts primary antioxidant systems, leading to decreased levels of reduced glutathione and increased levels of oxidized glutathione. This imbalance is also characterized by diminished superoxide dismutase activity and elevated lipid peroxidation	^88^
Β-ODAP	male and female mice	0.1 pM	inhibition of mitochondrial complex I and subsequent induction of oxidative stress	^89^
	M059 K cells	10 mM	increased Ca^2+^ influx and alterations in the microfilament structure of the neuronal cytoskeleton	^12^
	male Swiss albino mice	10 mg/kg	inhibition of catalase and glutathione peroxidase enzyme activity	^90^
DomA	primary mesencephalic cell cultures	0.1–100 μM	elevated levels of lactate dehydrogenase and destruction of dopaminergic neurons	^9^
	mouse cerebellar granule neurons	0.1–10 μM	elevated levels of ROS, increased oxidized glutathione, heightened intracellular Ca^2+^, activation of caspase-3, and reduced cell viability	^45^
l-HCA	mouse brain cortex	0.62–2.5 mM	elevated influx of Ca^2+^	^57^
	primary cortical neurons of mice	50 μM	hyperstimulation of NMDA receptors, increased Ca^2+^ influx, and generation of mitochondrial reactive oxygen species	^54^
l-Hcy	primary mouse cerebellum (postnatal days 30 and 60)	0.03 μmol/g body weight	the chronic hyperhomocysteinemia model induced oxidative stress and decreased mitochondrial complex IV activity	^91^

The elucidation of the mechanisms of neurotoxicity induced by l-Glu receptor agonists is essential for understanding the pathogenesis of neurodegenerative diseases. By interacting with NMDA, AMPA, KA receptors, l-Glu receptor agonists target the glutamatergic cycle, stimulating the synaptic release of l-Glu and disrupting Ca^2+^ homeostasis.^14,53^ This cascade of events leads to various deleterious outcomes, including mitochondrial dysfunction, increased oxidative stress, activation of proteolytic enzymes, and damage to cellular structures. Consequently, this can result in neuronal death through apoptosis or necrosis, contributing to the progression of the neurodegenerative processes. Excitotoxicity and the associated cellular damage are triggered by increased Ca^2+^ influx following the hyperstimulation of ionotropic channels by compounds structurally similar to l-Glu. Several nonproteinogenic amino acids, such as l-BMAA, β-ODAP, DomA, and l-HCA, possess chemical structures that facilitate interaction with l-Glu receptors and are linked to excitotoxicity.^18,61,67,68^ Additionally, metabolites of amino acids, such as QUIN, a derivative of l-trp, can also exhibit neurotoxic effects.^69^

l-Glutamate receptor agonists, whether natural or synthetic, can vary significantly in their affinity for different types of ionotropic glutamate receptors (iGluRs). This variation is highlighted by the concentration required for each agonist to produce 50% of the maximum receptor effect, known as the EC50 (50% effective concentration). Table 3 provides the EC_50_ values for l-Glu receptor agonists in relation to NMDA, AMPA, and kainate receptors. A lower EC50 value indicates that a smaller concentration of the agonist is needed to activate the receptor. Some agonists, such as l-BMAA, QUIN, and l-HCA, currently lack EC50 values, especially those concerning AMPA and kainate receptors. Similarly, β-ODAP does not have defined EC50 values, nor are there articles evaluating its direct activation of l-Glu receptors.^15,70–72^

**Table 3:** EC_50_ Values of Natural and Synthetic l-Glu Receptor Agonists for NMDA, AMPA, and Kainate Receptors

receptor compound		NMDA EC_50_ (μM)	AMPA EC_50_ (μM)	Kainato EC_50_ (μM)	reference
natural agonist	l-Glu	1.95–2.30	3.63–228.25	26.20	^70,71^
synthetic agonist	NMDA	34.90–38.32	n.f^a^	n.f	^70,71^
	AMPA	n.f	1.3–68.2	155.50	^70,71^
	Kainato	n.f	64.3–80.0	3.10	^70,71^
neurotoxins	l-BMAA	1000	n.f	n.f	^15^
	QUIN	400	n.f	n.f	^72^
	DomA	n.f	7.75	0.16	^70^
	Β-ODAP	n.f	n.f	n.f	-
	l-HCA	12.90–14.37	n.f	n.f	^70,71^

a Not found.

From the EC50 values in Table 3, we see that synthetic agonists like AMPA and kainate, despite their high specificity, might exhibit some functional overlap. Notably, l-BMAA and QUIN have the highest EC_50_ values for NMDA receptors, suggesting lower excitatory activity compared to l-glutamate. However, these compounds could still induce neurotoxicity through prolonged exposure. On the other hand, DomA shows the lowest EC_50_ value for kainate receptors, indicating higher potency at lower concentrations than the synthetic agonist itself. This explains its acute effects in documented food poisoning cases.^40,44^ For several agonists, quantitative studies or research focusing on individual receptors are still lacking. This limitation hampers meaningful comparisons and underscores the need for further investigations to fill this research gap.

Among the neurodegenerative diseases associated with excitotoxicity, ALS, neurolathyrism, and ASP are noteworthy. ALS is a progressive neurodegenerative disease that targets motor neurons, leading to muscle weakness and eventual paralysis. The association between l-BMAA and ALS was first identified in indigenous populations in Guam, where the incidence of the disease was significantly elevated. Studies suggest that this high prevalence may be linked to the bioaccumulation of l-BMAA in local dietary sources, such as cycad seeds and bats.^14,18^ Similarly, the cyclic amino acid β-ODAP, found in pea species of the genus Lathyrus, is another exogenous toxin associated with neurolathyrism. Chronic ingestion of this compound leads to compression of motor neurons, which can progress to irreversible paralysis of the lower limbs. *L. sativus* has long been utilized as a food source in regions with extreme climates, but the association with neurolathyrism has contributed to its reputation for toxicity.^12^

## Conclusion

In summary, excitotoxicity caused by l-glutamate and other agonists is a major focus in the development of new treatments for neurodegenerative diseases. This research particularly targets NMDA receptors due to their pivotal role in excitotoxic processes.^73^ Competitive and noncompetitive NMDA receptor antagonists, such as memantine, ketamine, and ifenprodil, have emerged as therapeutic strategies, despite their potential adverse effects. Furthermore, significant challenges such as BBB penetration, NMDA receptor diversity, bioavailability, discrepancies between preclinical and clinical trials, and the need to balance receptor inhibition without affecting their physiological functions in the CNS hinder the development of effective treatments.^60,74,75^

## Data Availability

Data for this work are archived and publicly available upon request at the authors.

## Abbreviations

^•^O_2_^–^: superoxide anion
^•^OH: hydroxyl radicals
3-HK: 3-hydroxykynurenine
ALS/PDC: amyotrophic lateral sclerosis Parkinson’s dementia complex
ALS: amyotrophic lateral sclerosis
AMPA: α-amino-3-hydroxy-5-methyl-4-isoxazolepropionic acid
ASP: amnesic shellfish poisoning
BBB: blood–brain barrier
Ca^2+^: calcium
CNS: central nervous system
Cyt *c*: cytochrome *c*
DomA: domoic acid
EAAT: excitatory amino acid transporters
H_2_O_2_: hydrogen peroxide
Hcy: homocysteine
IDO: indoleamine-2,3-dioxygenase
iGluRs: ionotropic glutamate receptors
INF-γ: interferon-gamma
K^+^: potassium
KA: kainite
KP: kynurenine pathway
KYNA: kynurenic acid
l-BMAA: β-*N*-methylamino-l-alanine
LDH: lactate dehydrogenase
l-Glu: l-glutamate
l-HCA: l-homocysteate
l-trp: l-tryptophan
mGluRs: metabotropic glutamate receptors
Na^+^: sodium
NAD^+^: nicotinamide adenine dinucleotide
NMDA: *N*-methyl-d-aspartate
PIC: picolinic acid
PKCα/βII: calcium-dependent protein kinases
QUIN: quinolinic acid
ROS: reactive oxygen species
SNAT: sodium-coupled neutral amino acid transporter
TCA: citric acid cycle
TDO: tryptophan-2,3-dioxygenase
TNF-α: tumor necrosis factor-alpha
β-ODAP: β-*N*-oxalyl-l-α,β-diaminopropionic acid
PDB: Protein Data Bank
